# High CD39 expression is associated with the non-muscle-invasive phenotype of human bladder cancer

**DOI:** 10.18632/oncotarget.28029

**Published:** 2021-08-03

**Authors:** Janaina Mendes Ferreira, Luiz Henrique Gomes Matheus, Renato Vasconcelos Souza de Almeida, Petronio Augusto de Souza Melo, Kátia Ramos Moreira Leite, Claudio Bovolenta Murta, Joaquim Francisco de Almeida Claro, Cleber Pinto Camacho, José Pontes-Júnior, Humberto Dellê

**Affiliations:** ^1^Postgraduate Program in Medicine, Universidade Nove de Julho, São Paulo, Brazil; ^2^Laboratory of Medical Investigation, Urology Department, University of São Paulo Medical School, São Paulo, Brazil; ^3^Divisão de Urologia do Centro de Saúde Masculina do Hospital Brigadeiro, São Paulo, Brazil

**Keywords:** bladder cancer, purinergic signaling, ectonucleotidases, CD39

## Abstract

Background: An accurate prediction of progression is critical to define the management of bladder cancer (BC). The ectonucleotidases CD39 and CD73 play strategic roles in calibrating purinergic signals via an extracellular balance between ATP and adenosine. The altered expression of these enzymes plays a potential role in tumor invasion and metastasis, therefore, has been proposed to be used for prognosis of solid tumor. In BC this is not yet clear.

Objective: This study aimed to evaluate CD39 and CD73 expression in a cohort of patients with non-muscle-invasive (NMI) and muscle-invasive (MI) BC regard to its association with clinicopathological features.

Materials and Methods: Retrospective clinical follow-up data and primary urothelial BC specimens of 162 patients were used (87 from patients who underwent transurethral resection and 75 from cystectomized patients). Tissue microarrays were constructed, and immunohistochemistry for CD39 and CD73 was performed to make associations with clinicopathological data.

Results: Overall, 96 were NMI (59.3%) and 66 MI (40.7%). CD39 immunoreactivity in BC cells was found in 72% of the cases, while CD73 was found in 97%. High CD39 expression alone was more frequent in NMI BC (*p* < 0.001), while CD73 expression was not powerful to predict the stage of BC. The association of both markers confirmed that only CD39 has potential in BC prognosis.

Conclusions: The altered expression of CD39 presented herein supports the idea that this ectonucleotidase may be involved in bladder tumorigenesis. High expression of CD39 in tumor cells is correlated with the early stage of BC.

## INTRODUCTION

Bladder cancer (BC) is the most common malignancy of the urinary tract, being the fourth most common cancer in men and the ninth in women [1]. Approximately 70% to 80% of BC cases are non-muscle-invasive (NMI), but a significant portion of those patients recurs and progresses to muscle-invasive (MI) form (10–25%) despite transurethral resection and adjuvant intravesical therapies [2]. MI BC is a more aggressive disease, being associated with a 5-years survival rate of 60% and 10% for patients with localized disease and metastases, respectively [2]. In these cases, radical cystectomy is often necessary. In this context, the management of BC and its complications results in a major economic burden.

Nucleotides and nucleosides constitute important groups of molecules known to modulate many pathophysiological functions in the extracellular space through activation of the purinergic receptors. The equilibrium of the purinergic signals is acquired by the conversion of ATP/ADP to AMP and AMP to adenosine, which occurs by hydrolytic enzymes [3]. One of these enzymes is the CD39 (ecto-nucleoside triphosphate diphosphohydrolase-1, NTPDase-1), an integral membrane protein that hydrolyzes ATP and ADP in order to yield AMP in the extracellular space [4]. The CD39 enzyme usually works with another enzyme named CD73 (ecto-5’-nucleotidase, Ecto5’NTase), which dephosphorylates AMP into adenosine [5]. While ATP drives pro-inflammatory immune cell activity, adenosine promotes a depressive action on the immune cell activity and has anti-inflammatory effects [3]. Therefore, the combination of CD39 and CD73 may determine an immunossuppressive environment to support neoplastic growth, survival, and progression [6]. Expression of CD39 and CD73 has been identified in various human tumors, including in kidney cancer, ovarian cancer, testicular cancer, pancreatic cancer, lung cancer, thyroid cancer, lymphoma, sarcoma, and leukemia [7–10]. Regarding BC, the role of CD39/CD73 is not clear, although purinergic signaling has been described in bladder tumors [11].

Stella et al. have shown that the profile of ectonucleotidase activity correlates to malignancy in BC. There is a distinct pattern in metabolizing nucleotides between NMI and MI bladder tumors. Working with human BC cell lines they demonstrated that BC cells with low malignancy phenotype established from a non-muscle invasive tumor exhibited a high level of hydrolysis of tri- and diphosphonucleosides due to high expression of NTPDase 3. In contrast, cells with a high malignancy phenotype derived from a muscle-invasive tumor exhibited a robust reduction in the NTPDase 3 expression [12]. A few years later the same group worked with a chemical-induced bladder tumor model demonstrating that the immunostaining for NTPDase is progressively lost during the disease progression, in contrast to CD73 expression that increased over time [13].

Considering the exposed above we hypothesized that information on the expression of CD39 and CD73 in human specimens of bladder tumor could be useful in the prognosis of BC. In the present study, we investigated the proteic expression of CD39 and CD73 in patients who underwent surgery for the treatment of NMI and MI urothelial bladder carcinoma.

## RESULTS

Of all 165 BC patients, 162 offered viable samples for CD39 and CD73 immunostaining analysis. The samples of three patients were deteriorated during the immunohistochemistry process. Of the 162 patients, 96 were NMI (59.3%) and 66 MI (40.7%). Overall, there were 129 males (79.6%) and 33 females (20.4%) in this cohort. Most were elderly (70.4% > 65 years old), and smokers (79.5%) (Table 1). Of the 162 tumor specimens, 87 (53.7%) were obtained from TUR, and 75 (46.3%) were obtained from cystectomy (Table 1). Regarding histological grade, 74% of patients were high grade and 26% were low grade. The tumor stage between pTa to pT4 was found in our cohort, with pTa being more prevalent (46.8%). Of all 87 patients undergoing TUR for a primary pTa or pT1, 50 had tumor recurrence (57.5%, average time of 39 months). Progression disease was also evaluated in patients who underwent TUR, 14 of whom had disease progression (16.1%) (Table 1). Survival and disease-free survival were analyzed, thus no difference was observed between groups.

**Table 1:** Correlation of CD39 expression with clinicopathological parameters of 162 BC patients

Parameters	No. of Patients	CD39			*P* Value
		Negative	Low expression	High expression	
Gender					
Male	129	39 (30%)	34 (26%)	56 (44%)	*0.538*
Female	33	07 (21%)	11 (33%)	15 (46%)	
Age					
≤ 65	48	13 (27%)	12 (25%)	23 (48%)	*0.782*
> 65	114	33 (29%)	33 (29%)	48 (42%)	
Smoking					
No	31	06 (19%)	06 (19%)	19 (61%)	*0.128*
Yes	120	35 (29%)	35 (29%)	50 (42%)	
Unknown	11	05 (46%)	04 (36%)	02 (18%)	
Grade					
Low	42	8 (19%)	16 (38%)	18 (43%)	*0.140*
High	120	38 (32%)	29 (24%)	53 (44%)	
Tumor stage					
NMI	96	11 (11%)	35 (36%)	50 (52%)	***<0.001***
MI	66	35 (53%)	10 (15%)	21 (32%)	
Tumor stage					
pTa	71	09 (13%)	24 (34%)	38 (54%)	***<0.001***
pT1	25	02 (08%)	11 (44%)	12 (48%)	
pT2	21	14 (67%)	02 (10%)	05 (24%)	
pT3	26	11 (42%)	05 (19%)	10 (39%)	
pT4	19	10 (53%)	03 (16%)	06 (32%)	
Treatment					
TUR	87	09 (10%)	32 (37%)	46 (53%)	***<0.001***
Cystectomy	75	37 (49%)	13 (17%)	25 (33%)	
Recurrence^*^					
No	37	04 (11%)	14 (38%)	19 (51%)	*0.970*
Yes	50	05 (10%)	18 (36%)	27 (54%)	
Progression^*^					
No	73	09 (12%)	28 (38%)	36 (49%)	*0.210*
Yes	14	02 (14%)	02 (14%)	10 (71%)	

^*^Only patients who underwent transurethral resection (TUR).

**Figure 1:**
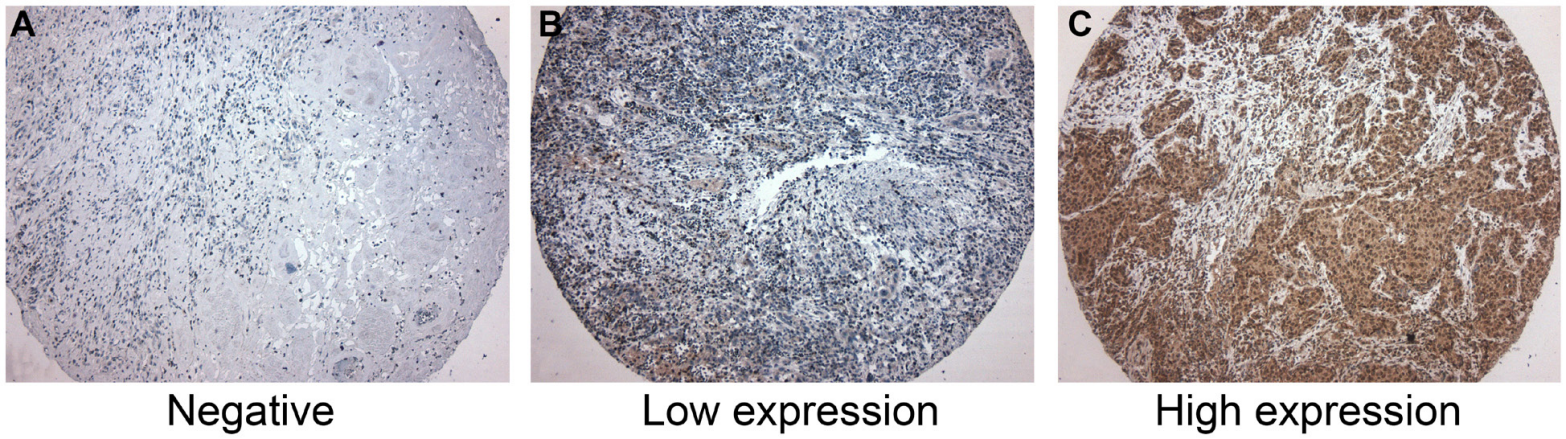
Immunohistochemistry for CD39. (**A**) Negative for CD39 expression. (**B**) Low CD39 expression. Few neoplastic cells were positive for CD39. (**C**) High CD39 expression, showing more than 50% of positive cells for CD39. CD39 expression was predominantly cytoplasmatic in neoplastic cells. CD39-expressing inflammatory cells were not considered in this study.

All tumor samples could be evaluated for CD39 and CD73 immunostaining (three TMA cores per patient). The immunoreactivity for both CD39 and CD73 was predominantly cytoplasmatic in neoplastic cells (Figure 1). Of the total number of patients, 46 (28.4%) were negative for CD39 immunostaining, 45 (27.8%) presented weak CD39 immunostaining (low expression), and 71 (43.8%) presented strong CD39 immunostaining (high expression). As demonstrated in Table 1, high CD39 expression was associated with NMI (*P* < 0.001), and lower tumor stage (*p* < 0.001). High CD39 expression was also associated with the modality of treatment (TUR), probably due to the association between the high CD39 expression and NMI BC. CD39 expression was not associated with grade, recurrence, or tumor progression (Table 1).

Regarding CD73 immunostaining, five patient samples showed no expression, 24 showed low expression of CD73, while 133 showed high expression (Supplementary Table 1). CD73 immunostaining was not associated with clinicopathological features. When combined to the CD39 expression, a significant association with tumor stage was found, but CD73 expression was indifferent in this prediction (Supplementary Table 2). Low expression of both CD39 and CD73 was associated with advanced BC, similarly to the condition of low expression of CD39 and high expression of CD73 (Supplementary Table 2). In contrast, high expression of CD39 and low expression of CD73 as well as the high expression for both proteins was associated with the NMI form of BC (Supplementary Table 2).

## DISCUSSION

The ectonucleotidase enzymes play distinct roles in mammalian organisms by regulating the extracellular concentration of ATP/ADP/AMP/adenosine. The balance of nucleotides/adenosine determines the activation of distinct purinergic receptors triggering specific effects. Like other pleiotropic molecules, the expression of these enzymes may be found in several normal tissues. CD39 and CD73 are constitutively expressed in a variety of tissues and leukocytes and may be upregulated by pro-inflammatory cytokines, hypoxia, and oxidative stress [3]. Regarding normal bladder, the ectonucleotidases play role in nerve-mediated detrusor contractions and in exocytosis of the umbrella cell layer by controlling the ATP/adenosine concentrations in the extracellular space [14, 15]. It is possible that CD39 and CD73 are involved in other physiological processes of the lower urinary tract, but this remains uncertain.

In the pathophysiological context, CD39 and CD73 have been found in neoplasias raising the hypothesis of their involvement in tumorigenesis [7, 16]. In the present study, we have shown that the expression ratio of CD39/CD73 is associated with the non-muscle-invasive form of BC, raising the hypothesis that the nucleotides/adenosine balance may be effective in determining invasive phenotype in BC cells.

With regard to CD39, its expression has not been studied in BC until now. On the other hand, there is some information about CD73 in urothelial tumors. Wettstein and coworkers showed that CD73 predicted favorable prognosis in patients with NMI urothelial BC [17]. In their study, cases of high expression of CD73 were more frequent in pT1 and pTa stages, although no association with CD39 expression was made [17]. More recently, Koivisto and coworkers demonstrated similar results, expanding the analysis to MI BC. They demonstrated that high expression of CD73 by neoplastic cells was more frequent in NMI than in MI BC [18], a phenomenon not found in our study. In our study, isolated CD73 expression was not sufficient to predict the BC stage, suggesting the use of isolated CD39 expression or CD39/CD73 expression balance as a useful strategy to assess the invasive status of the BC. In other tumors, CD73 has been associated with the worst prognosis. CD73 overexpression promotes invasion, migration, adhesion, and metastasis of human breast cancer cells and in melanomas [19, 20].

Although there is little information about CD39 in BC, it has been widely described in other tumor types. CD39 is overexpressed in pancreatic cancer correlating positively with long-term survival after surgery treatment [21]. In rectal adenocarcinoma, although CD39 was strongly expressed in malignant cells being associated with early tumor stage, its association with CD73 offered a better strategy to predict the prognosis [22]. The combined analysis displaying low CD39 and high CD73 expression in both protein and mRNA levels was associated with invasion and metastasis leading to an unfavorable clinical outcome [22], similarly as we have proposed herein for BC.

In terms of mechanism, acting sequentially, CD39 and CD73 efficiently hydrolyze extracellular ATP to adenosine, the latter having an effect in down-modulating the antitumor immunity via binding to purinergic receptors. Adenosine-activated A2A receptor protects tumors from antitumor T cells [23]. The blockage of CD73 using a monoclonal antibody enhances antitumor response [24]. Different from adenosine, ATP is a potent pro-inflammatory mediator limiting tumor growth. To promote antitumor immunity, ATP activates inflammasome [25], and pyroptosis [26], promotes chemotaxis of monocytes, macrophages, and neutrophils [27], and controls tumor-infiltrating CD8+ T cells [28] and NK cells [29].

Considering the above, we would propose that the overexpression of CD39 and/or CD73 could increase the adenosine concentration in the extracellular space, which could induce local immunosuppression and consequently tumor progression. However, cases of CD39_Low_CD73_Low_ and CD39_High_CD73_High_ were more frequent in MI and NMI BC, respectively. In fact, access to ATP/adenosine status would be critical in understanding this controversial phenomenon. An important limitation of our study is the lack of technical approaches to determine ATP/adenosine in patient tumor specimens. Immunostaining analysis for CD39 and CD73 is a simplistic form to access the complex nucleotides/adenosine system. The final effects will be determined by multiple factors. First, the amount of ATP that moves into the extracellular space, either through cell injury or through channel transport. The tumor microenvironment can contain a 1000-fold higher ATP concentration in the interstitial space compared to normal tissues [6]. Second, in addition to CD39 and CD73, both nucleotides and nucleosides can be removed from extracellular space by several other ectoenzyme families [5]. Third, ATP and adenosine exert effects by binding purinergic receptors - P1 receptors, with adenosine as the main ligand, and P2 receptors, with ATP and ADP as the main ligands; P2 receptors are subdivided into seven ionotropic P2X and eight metabotropic P2Y subtypes [30, 31]. Therefore, the roles of ATP/adenosine balance in BC will depend on various expression levels and coexpression patterns.

Finally, our study complements the findings of Stella and coworkers, in which the profile characterized by downregulation of NTPDase 3 and upregulation of CD73 was associated with the late stage of BC [12], a phenomenon also demonstrated in an animal model [13]. In their hypothesis, that profile establishes an extracellular microenvironment with high levels of ATP and adenosine [12] favorable to tumor progression [12].

In conclusion, the altered expression of CD39 presented herein supports the idea that this ectonucleotidase may be involved in bladder tumorigenesis. Our results suggest that malignant urothelial cells of human BC strongly express CD39; however, high expression of CD39 in tumor cells is correlated with the early stage of BC. Further studies are necessary to clarify the effective role of loss/downregulation of CD39 expression in the establishment of invasive phenotype in BC cells.

## MATERIALS AND METHODS

Retrospective clinical follow-up data and tumor specimens of 165 patients were used in this study. Our cohort is represented by a series of 165 consecutive primary urothelial bladder tumors consisting of pTa, pT1, pT2, pT3, and pT4 collected between 2011 and 2017 who underwent surgery. The samples were made available by the Department of Pathology of the Hospital Brigadeiro where the patients underwent transurethral resection (TUR) or radical cystectomy. Tumor stage and grade were assigned according to AJCC/TNM 2010 and WHO 2004 classifications. CB recurrence or metastasis was determined via imaging and/or histological analysis. The descriptive characteristics of the cohort are depicted in Table 1. The exclusion criteria were: (i) patients with the previous history of other cancers; (ii) patients had received preoperative chemotherapy or radiation therapy; and (iii) unavailability of clinicopathological data or paraffin blocks. Approval for the study was given by the Institutional Board of Ethics of the Hospital Brigadeiro UGA V-SP endorsed by the Institutional Board of Ethics of the Nove de Julho University (UNINOVE) (CAAE: 49446515.0.0000.5511).

Tissue microarrays (TMA) were constructed from 165 formalin-fixed, paraffin-embedded surgical specimens (from TUR or cystectomy), represented in triplicate tissue cores of 1-millimeter diameter. An experienced single uro-pathologist reevaluated the hematoxylin-and-eosin-stained slides of all specimens and considered the areas that best represented the whole tumor.

### Immunohistochemistry

TMA was cut and used on 3-μm-thick paraffin sections. After rehydration, antigen retrieval (microwave irradiation in citrate buffer), and blocking procedures, the sections were incubated overnight with mouse monoclonal anti-human CD39 antibody (1:500; catalog ab178572; Abcam, Cambridge, MA, USA) or rabbit polyclonal anti-human CD73 antibody (1:50; catalog ab115289; Abcam, Cambridge, MA, USA). The negative control was performed omitting the primary antibodies. To complete the sandwich, sections were incubated with LSAB+ System-HRP reagents (K0690; Dako Co., Glostrup, Denmark). Finally, DAB substrate-chromogen was used to complete the reaction (K346811; Dako Co., Glostrup, Denmark), and the slides were counterstained with Harris hematoxylin. Slides were then dehydrated, coverslipped, and observed under a light microscope.

The expression of CD39 and CD73 was evaluated blindly by a single uro-pathologist (KRML) who employed a semiquantitative classification where the proportion score reflects the percentage of tumor cells with CD39 or CD73 staining: score 0, lack of expression; score 1, positive tumor area < 50% (low expression); and score 2, positive area > 50% (high expression). As the TMA was carried out in triplicate, the higher value was considered. For statistical analysis, were formed three groups: absent expression, low expression, and high expression, according to the score described above. Alternatively, to divide all cases into only two groups (low and high expression, as represented in Table 2), a cut-off was used. Briefly, the scores of each patient were summed and the median was used as the cut-off.

**Table 2:** Expression of CD39 and CD73 in the prediction of BC stage

	Odds Ratios	95% CI	*P*-value	Prediction of
CD39_High_	0.158	0.079–0.315	***<0.001***	**NMI**
CD73_High_	1.077	0.570–2.034	*0.872*	**None**
CD39_Low_CD73_Low_	2.520	1.183–5.368	***0.013***	**MI**
CD39_High_CD73_Low_	0.269	0.104–0.698	***0.005***	**NMI**
CD39_Low_CD73_High_	5.884	2.432–14.232	***<0.001***	**MI**
CD39_High_CD73_High_	0.334	0.167–0.666	***0.002***	**NMI**

Abbreviations: NMI, non-muscle-invasive; MI, muscle-invasive.

### Statistical analysis

Pearson’s Chi-square test, Fisher’s exact test, and logistic regression were used to assess the association between clinical-pathological features and CD39 and CD73 expression. The odds ratio was also evaluated, in which a confidence interval of 95% was adopted. The analysis was performed using SPSS (version 22; SPSS, Inc., Chicago, IL), the same program for graphing. Differences with *p* < 0.05 were considered significant.

## SUPPLEMENTARY MATERIALS



## Abbreviations

BC: bladder cancer
NMI: non-muscle-invasive
MI: muscle-invasive
TMA: Tissue microarray

## References

[1] Antoni S , Ferlay J , Soerjomataram I , Znaor A , Jemal A , Bray F . Bladder Cancer Incidence and Mortality: A Global Overview and Recent Trends. Eur Urol. 2017; 71:96–108. 10.1016/j.eururo.2016.06.010. 27370177

[2] Kamoun A , de Reyniès A , Allory Y , Sjödahl G , Robertson AG , Seiler R , Hoadley KA , Groeneveld CS , Al-Ahmadie H , Choi W , Castro MAA , Fontugne J , Eriksson P , et al. A Consensus Molecular Classification of Muscle-invasive Bladder Cancer. Eur Urol. 2020; 77:420–33. 10.1016/j.eururo.2019.09.006. 31563503 PMC7690647

[3] Antonioli L , Pacher P , Vizi ES , Haskó G . CD39 and CD73 in immunity and inflammation. Trends Mol Med. 2013; 19:355–67. 10.1016/j.molmed.2013.03.005. 23601906 PMC3674206

[4] Heine P , Braun N , Sévigny J , Robson SC , Servos J , Zimmermann H . The C-terminal cysteine-rich region dictates specific catalytic properties in chimeras of the ectonucleotidases NTPDase1 and NTPDase2. Eur J Biochem. 2001; 268:364–73. 10.1046/j.1432-1033.2001.01896.x. 11168371

[5] Yegutkin GG . Enzymes involved in metabolism of extracellular nucleotides and nucleosides: functional implications and measurement of activities. Crit Rev Biochem Mol Biol. 2014; 49:473–97. 10.3109/10409238.2014.953627. 25418535

[6] Di Virgilio F , Adinolfi E . Extracellular purines, purinergic receptors and tumor growth. Oncogene. 2017; 36:293–303. 10.1038/onc.2016.206. 27321181 PMC5269532

[7] Bastid J , Regairaz A , Bonnefoy N , Déjou C , Giustiniani J , Laheurte C , Cochaud S , Laprevotte E , Funck-Brentano E , Hemon P , Gros L , Bec N , Larroque C , et al. Inhibition of CD39 enzymatic function at the surface of tumor cells alleviates their immunosuppressive activity. Cancer Immunol Res. 2015; 3:254–65. 10.1158/2326-6066.CIR-14-0018. 25403716

[8] Hayes GM , Cairns B , Levashova Z , Chinn L , Perez M , Theunissen JW , Liao-Chan S , Bermudez A , Flory MR , Schweighofer KJ , van der Horst EH . CD39 is a promising therapeutic antibody target for the treatment of soft tissue sarcoma. Am J Transl Res. 2015; 7:1181–88. 26279761 PMC4532750

[9] Pulte D , Furman RR , Broekman MJ , Drosopoulos JH , Ballard HS , Olson KE , Kizer JR , Marcus AJ . CD39 expression on T lymphocytes correlates with severity of disease in patients with chronic lymphocytic leukemia. Clin Lymphoma Myeloma Leuk. 2011; 11:367–72. 10.1016/j.clml.2011.06.005. 21816376 PMC3590911

[10] Häusler SF , Montalbán del Barrio I , Strohschein J , Chandran PA , Engel JB , Hönig A , Ossadnik M , Horn E , Fischer B , Krockenberger M , Heuer S , Seida AA , Junker M , et al. Ectonucleotidases CD39 and CD73 on OVCA cells are potent adenosine-generating enzymes responsible for adenosine receptor 2A-dependent suppression of T cell function and NK cell cytotoxicity. Cancer Immunol Immunother. 2011; 60:1405–18. 10.1007/s00262-011-1040-4. 21638125 PMC11028787

[11] Shabbir M , Ryten M , Thompson C , Mikhailidis D , Burnstock G . Characterization of calcium-independent purinergic receptor-mediated apoptosis in hormone-refractory prostate cancer. BJU Int. 2008; 101:352–59. 10.1111/j.1464-410x.2007.07293.x. 18005209

[12] Stella J , Bavaresco L , Braganhol E , Rockenbach L , Farias PF , Wink MR , Azambuja AA , Barrios CH , Morrone FB , Oliveira Battastini AM . Differential ectonucleotidase expression in human bladder cancer cell lines. Urol Oncol. 2010; 28:260–67. 10.1016/j.urolonc.2009.01.035. 19372055

[13] Rockenbach L , Braganhol E , Dietrich F , Figueiró F , Pugliese M , Edelweiss MI , Morrone FB , Sévigny J , Battastini AM . NTPDase3 and ecto-5'-nucleotidase/CD73 are differentially expressed during mouse bladder cancer progression. Purinergic Signal. 2014; 10:421–30. 10.1007/s11302-014-9405-8. 24464643 PMC4152458

[14] McCarthy CJ , Ikeda Y , Skennerton D , Chakrabarty B , Kanai AJ , Jabr RI , Fry CH . Characterisation of nerve-mediated ATP release from bladder detrusor muscle and its pathological implications. Br J Pharmacol. 2019; 176:4720–30. 10.1111/bph.14840. 31430833 PMC6965683

[15] Wang EC , Lee JM , Ruiz WG , Balestreire EM , von Bodungen M , Barrick S , Cockayne DA , Birder LA , Apodaca G . ATP and purinergic receptor-dependent membrane traffic in bladder umbrella cells. J Clin Invest. 2005; 115:2412–22. 10.1172/jci24086. 16110327 PMC1187935

[16] Zhang B . CD73 promotes tumor growth and metastasis. Oncoimmunology. 2012; 1:67–70. 10.4161/onci.1.1.18068. 22720214 PMC3376970

[17] Wettstein MS , Buser L , Hermanns T , Roudnicky F , Eberli D , Baumeister P , Sulser T , Wild P , Poyet C . CD73 Predicts Favorable Prognosis in Patients with Nonmuscle-Invasive Urothelial Bladder Cancer. Dis Markers. 2015; 2015:785461. 10.1155/2015/785461. 26543299 PMC4620269

[18] Koivisto MK , Tervahartiala M , Kenessey I , Jalkanen S , Boström PJ , Salmi M . Cell-type-specific CD73 expression is an independent prognostic factor in bladder cancer. Carcinogenesis. 2019; 40:84–92. 10.1093/carcin/bgy154. 30395172

[19] Stagg J , Divisekera U , McLaughlin N , Sharkey J , Pommey S , Denoyer D , Dwyer KM , Smyth MJ . Anti-CD73 antibody therapy inhibits breast tumor growth and metastasis. Proc Natl Acad Sci U S A. 2010; 107:1547–52. 10.1073/pnas.0908801107. 20080644 PMC2824381

[20] Stagg J , Divisekera U , Duret H , Sparwasser T , Teng MW , Darcy PK , Smyth MJ . CD73-deficient mice have increased antitumor immunity and are resistant to experimental metastasis. Cancer Res. 2011; 71:2892–900. 10.1158/0008-5472.CAN-10-4246. 21292811

[21] Künzli BM , Berberat PO , Giese T , Csizmadia E , Kaczmarek E , Baker C , Halaceli I , Büchler MW , Friess H , Robson SC . Upregulation of CD39/NTPDases and P2 receptors in human pancreatic disease. Am J Physiol Gastrointest Liver Physiol. 2007; 292:G223–30. 10.1152/ajpgi.00259.2006. 16920697

[22] Zhang B , Cheng B , Li FS , Ding JH , Feng YY , Zhuo GZ , Wei HF , Zhao K . High expression of CD39/ENTPD1 in malignant epithelial cells of human rectal adenocarcinoma. Tumour Biol. 2015; 36:9411–19. 10.1007/s13277-015-3683-9. 26113408

[23] Ohta A , Gorelik E , Prasad SJ , Ronchese F , Lukashev D , Wong MK , Huang X , Caldwell S , Liu K , Smith P , Chen JF , Jackson EK , Apasov S , et al. A2A adenosine receptor protects tumors from antitumor T cells. Proc Natl Acad Sci U S A. 2006; 103:13132–37. 10.1073/pnas.0605251103. 16916931 PMC1559765

[24] Allard B , Pommey S , Smyth MJ , Stagg J . Targeting CD73 enhances the antitumor activity of anti-PD-1 and anti-CTLA-4 mAbs. Clin Cancer Res. 2013; 19:5626–35. 10.1158/1078-0432.CCR-13-0545. 23983257

[25] Gaidt MM , Hornung V . The NLRP3 Inflammasome Renders Cell Death Pro-inflammatory. J Mol Biol. 2018; 430:133–41. 10.1016/j.jmb.2017.11.013. 29203171

[26] Taylor SR , Gonzalez-Begne M , Dewhurst S , Chimini G , Higgins CF , Melvin JE , Elliott JI . Sequential shrinkage and swelling underlie P2X7-stimulated lymphocyte phosphatidylserine exposure and death. J Immunol. 2008; 180:300–08. 10.4049/jimmunol.180.1.300. 18097031

[27] Elliott MR , Chekeni FB , Trampont PC , Lazarowski ER , Kadl A , Walk SF , Park D , Woodson RI , Ostankovich M , Sharma P , Lysiak JJ , Harden TK , Leitinger N , Ravichandran KS . Nucleotides released by apoptotic cells act as a find-me signal to promote phagocytic clearance. Nature. 2009; 461:282–86. 10.1038/nature08296. 19741708 PMC2851546

[28] Canale FP , Ramello MC , Núñez N , Araujo Furlan CL , Bossio SN , Gorosito Serrán M , Tosello Boari J , Del Castillo A , Ledesma M , Sedlik C , Piaggio E , Gruppi A , Acosta Rodríguez EA , Montes CL . CD39 Expression Defines Cell Exhaustion in Tumor-Infiltrating CD8^+^ T Cells. Cancer Res. 2018; 78:115–28. 10.1158/0008-5472.CAN-16-2684. 29066514

[29] Zhang H , Vijayan D , Li XY , Robson SC , Geetha N , Teng MWL , Smyth MJ . The role of NK cells and CD39 in the immunological control of tumor metastases. Oncoimmunology. 2019; 8:e1593809. 10.1080/2162402X.2019.1593809. 31069159 PMC6492967

[30] Ralevic V , Burnstock G . Receptors for purines and pyrimidines. Pharmacol Rev. 1998; 50:413–92. 9755289

[31] White N , Burnstock G . P2 receptors and cancer. Trends Pharmacol Sci. 2006; 27:211–17. 10.1016/j.tips.2006.02.004. 16530853

