# Supplementary material for: High CD39 expression is associated with the non-muscle-invasive phenotype of human bladder cancer
Source: Oncotarget. 2021 Aug 3;12(16):1580–6. doi: 10.18632/oncotarget.28029 (PMC8351603; doi:10.18632/oncotarget.28029)
Supplement: Supplementary file 1 [file oncotarget-12-1580-s001.pdf]

High CD39 expression is associated with the non-muscle-invasive phenotype of human bladder cancer

SUPPLEMENTARY MATERIALS

Supplementary Table 1: Correlation of CD73 expression with clinicopathological parameters of 162 BC patients

Parameters	No. of Patients	CD73			*P* Value
		Negative	Low expression	High expression	
Gender					
Male	129	04 (03%)	22 (17%)	103 (80%)	*0.281*
Female	33	01 (03%)	02 (06%)	30 (91%)	
Age					
≤ 65	48	00 (00%)	06 (13%)	42 (87%)	*0.273*
> 65	114	05 (04%)	18 (16%)	91 (80%)	
Smoking					
No	31	01 (03%)	06 (19%)	24 (78%)	*0.619*
Yes	120	04 (03%)	15 (13%)	101 (84%)	
Unknown	11	00 (00%)	03 (27%)	08 (73%)	
Grade					
Low	42	01 (02%)	06 (14%)	35 (34%)	*0.945*
High	120	04 (03%)	18 (15%)	98 (82%)	
Tumor stage					
NMI	96	03 (03%)	13 (14%)	80 (83%)	*0.860*
MI	66	02 (03%)	11 (17%)	53 (80%)	
Tumor stage					
pTa	71	03 (04%)	09 (13%)	59 (83%)	*0.612*
pT1	25	00 (00%)	04 (16%)	21 (84%)	
pT2	21	01 (05%)	04 (19%)	16 (76%)	
pT3	26	00 (00%)	02 (19%)	24 (92%)	
pT4	19	01 (05%)	05 (26%)	13 (69%)	
Treatment					
TUR	87	02 (02%)	12 (14%)	73 (84%)	*0.746*
Cystectomy	75	03 (04%)	12 (16%)	60 (80%)	
Recurrence*					
No	37	01 (03%)	05 (14%)	31 (84%)	*0.976*
Yes	50	01 (02%)	07 (14%)	42 (84%)	
Progression*					
No	73	02 (03%)	09 (12%)	62 (85%)	*0.564*
Yes	14	00 (00%)	03 (21%)	11 (79%)	

*Only patients who underwent transurethral resection (TUR).

Supplementary Table 2: Correlation of different profiles of CD39 and CD73 expression with clinicopathological parameters of 162 BC patients

Parameters	No. of Patients	CD39-CD73				*P* Value
		$CD39_{Low}CD73_{Low}$	$CD39_{High}CD73_{Low}$	$CD39_{Low}CD73_{High}$	$CD39_{High}CD73_{High}$	
Gender						
Male	129	30 (23%)	28 (22%)	24 (19%)	47 (36%)	*0.457*
Female	33	06 (18%)	04 (12%)	07 (21%)	16 (49%)	
Age						
≤ 65	48	09 (19%)	11 (23%)	10 (21%)	18 (38%)	*0.837*
> 65	114	27 (24%)	21 (18%)	21 (18%)	45 (40%)	
Smoking						
No	31	06 (19%)	12 (39%)	02 (07%)	11 (36%)	***0.007***
Yes	120	25 (21%)	19 (16%)	25 (21%)	51 (43%)	
Unknown	11	05 (46%)	01 (09%)	04 (36%)	01 (09%)	
Grade						
Low	42	08 (19%)	12 (29%)	05 (12%)	17 (41%)	*0.253*
High	120	28 (23%)	20 (17%)	26 (22%)	46 (38%)	
Tumor stage						
NMI	96	15 (16%)	26 (27%)	08 (08%)	47 (49%)	***<0.001***
MI	66	21 (32%)	06 (09%)	23 (35%)	16 (24%)	
Tumor stage						
pTa	71	12 (17%)	22 (31%)	05 (07%)	32 (45%)	***<0.001***
pT1	25	03 (12%)	04 (16%)	03 (12%)	15 (60%)	
pT2	21	09 (43%)	02 (09%)	05 (24%)	05 (24%)	
pT3	26	07 (27%)	01 (04%)	10 (39%)	08 (31%)	
pT4	19	05 (26%)	03 (16%)	08 (42%)	03 (16%)	
Treatment						
TUR	87	12 (14%)	26 (30%)	06 (07%)	43 (49%)	***<0.001***
Cystectomy	75	24 (32%)	06 (08%)	25 (33%)	20 (27%)	
Recurrence*						
No	37	08 (22%)	09 (24%)	02 (05%)	18 (49%)	*0.291*
Yes	50	04 (08%)	17 (34%)	04 (08%)	25 (50%)	
Progression*						
No	73	11 (15%)	21 (29%)	05 (07%)	36 (49%)	*0.867*
Yes	14	01 (07%)	05 (36%)	01 (07%)	07 (50%)	

*Only patients who underwent transurethral resection (TUR).
